# Notes, Clinical and Pathogenetic

**Published:** 1885-12

**Authors:** E. W. Berridge

**Affiliations:** London


### NOTES, CLINICAL AND PATHOGENETIC.

E. W. Berridge, M. D., London.

1. *Kali carb.*—Mr. W., after taking *Kali carb.*$^{3cm}$ (Fincke) every two hours for acute spinal disease, noticed the following symptoms: dreamed the same dream repeatedly, about a dozen times in all, and on waking had a vivid recollection of it; after he told his wife it did not return. Compare similar symptoms produced in another patient of *Kali carb.*$^{4m}$ (Jenichen), numbered 1629 in Allen's *Encyclopædia.*

2. *Kali carb.*—Miss H. took for myelitis *Kali carb.*$^{30}$, 3cm (Fincke) and cm (F. C.) with great benefit, but preceded by aggravation; the 3cm caused most aggravation, the 30 and the cm least of all.

3. *Podophyllum.*—Mr. J. once took, by the advice of a pseudo-homœopath, a drop of the strong tincture of *Podophyllum* in the evening. Next morning there was dryness and swelling of tongue, which was yellow-striped; in two or three days more the tongue become quite red and very rough, still remaining dry and swelled. These symptoms lasted quite two weeks. On two other occasions a dose produced similar effects on him, in one instance lasting ten days.

4. *Zinc.*—Miss S., æt. fourteen, suffering from left hip disease, was much relieved by *Zincum* $^{cm}$ (Fincke), the keynote of the case being "*cracking in sacral region,*" which was removed by the medicine. See Bœnninghausen's *Repertory*, p. 170.

5. *Hyoscyamus.*—Mrs. B. was delivered at twenty-five minutes past twelve P. M. At nine P. M. complained that she had passed no urine since half-past nine A. M. Has almost constant desire to urinate with discomfort in bladder, aggravated by the least movement, and by lying on either side, relieved by lying on back; putting the child to the breast caused the same symptoms. *Hyoscyamus* $^{1m}$ (Jenichen), promptly cured.

6. *Saccharum lactis.*—Mrs. B. felt thoroughly exhausted from overwork and nursing a patient. Gave a dose of *Sacch. lactis* $^{cm}$ (Fincke), *and in five minutes the exhaustion was gone.* (This has been verified by others.)

